# Hand infections in diabetics: risk factors

**DOI:** 10.1186/1753-6561-9-S3-A101

**Published:** 2015-05-19

**Authors:** Nathaniel Orillaza

**Affiliations:** 1 University of the Philippines, Manila, Philippines

## 

Hand Infection in diabetics, or sometimes referred to as Tropical Diabetic Hand Syndrome (TDHS), is still a problem in developing countries around the world. While the incidence may be going down in developed countries, reports from less developed communities are still concerning.

Demographics vary between centers. While the incidence is a lot less than leg and foot infections, the consequences of delayed treatment and poor infection control are worse in terms of function and, in some reports, even in length and quality of life.

The exact pathogenesis, including predisposing factors of TDHS, is still poorly understood. The role of impaired perfusion and nerve function and being immunocompromised through different mechanisms are commonly described reasons for predisposing diabetic patients to infection. Several attempts to identify specific causative factors in patient and treatment variables are promising but have shown inconsistent findings.

Lesions may range from cellulitis, to abscesses (including tenosynovitis) to osteomyelitis and gangrene. There have been a good number of published articles on TDHS but most are small series of patients with infections requiring admission and surgical management. In a larger series including consults at the clinics, it would seem that most cases of infection are still relatively benign and only required medical management. Coverage for polymicrobial involvement, including gram positive and gram negatives, anaerobes and sometimes fungi, should be considered when giving antibiotics.

Patients requiring admission had problems related to other complications of diabetes and/or more fulminant infections. Debridement is still the most common procedure while amputation rates vary significantly between institutions. It is generally accepted that surgical management, if needed, should be done early.

Our own series of patients showed a similar population in age compared to other studies, with more severe infections at presentation. We have a higher amputation rate compared to literature and comparing against controls (diabetic patients with no hand infection), uncontrolled hyperglycemia and tobacco use appeared to be important risk factors in developing infection.

